# Exploring Environmental Factors in Nursing Workplaces That Promote Psychological Resilience: Constructing a Unified Theoretical Model

**DOI:** 10.3389/fpsyg.2016.00600

**Published:** 2016-05-13

**Authors:** Lynette Cusack, Morgan Smith, Desley Hegney, Clare S. Rees, Lauren J. Breen, Regina R. Witt, Cath Rogers, Allison Williams, Wendy Cross, Kin Cheung

**Affiliations:** ^1^Faculty Health Sciences, School of Nursing, University of AdelaideAdelaide, SA, Australia; ^2^School of Nursing and Midwifery, The University of Southern QueenslandToowoomba, QLD, Australia; ^3^School of Psychology and Speech Pathology, Faculty of Health Sciences, Curtin UniversityPerth, WA, Australia; ^4^Nursing School, Universidade Federal do Rio Grande do SulPorto Alegre, Brazil; ^5^School of Nursing and Midwifery, Monash UniversityClayton, VIC, Australia; ^6^School of Nursing, The Hong Kong Polytechnic UniversityHonk Kong, China

**Keywords:** resilience, nurses, workplace, environment

## Abstract

Building nurses' resilience to complex and stressful practice environments is necessary to keep skilled nurses in the workplace and ensuring safe patient care. A unified theoretical framework titled Health Services Workplace Environmental Resilience Model (HSWERM), is presented to explain the environmental factors in the workplace that promote nurses' resilience. The framework builds on a previously-published theoretical model of individual resilience, which identified the key constructs of psychological resilience as self-efficacy, coping and mindfulness, but did not examine environmental factors in the workplace that promote nurses' resilience. This unified theoretical framework was developed using a literary synthesis drawing on data from international studies and literature reviews on the nursing workforce in hospitals. The most frequent workplace environmental factors were identified, extracted and clustered in alignment with key constructs for psychological resilience. Six major organizational concepts emerged that related to a positive resilience-building workplace and formed the foundation of the theoretical model. Three concepts related to nursing staff support (professional, practice, personal) and three related to nursing staff development (professional, practice, personal) within the workplace environment. The unified theoretical model incorporates these concepts within the workplace context, linking to the nurse, and then impacting on personal resilience and workplace outcomes, and its use has the potential to increase staff retention and quality of patient care.

## Introduction

The purpose of this paper is to explain theoretically the environmental factors that affect nurses' resilience in the workplace. This research builds on a previous theoretical model of workplace psychological resilience developed by Rees et al. (2015). Understanding the environmental factors in the workplace that are essential to promote the psychological resilience of nurses is important to the health care system due to the potential to increase staff retention, whilst reducing workplace distress, burnout and compassion fatigue, and enhancing patient safety (Lowe, 2013). Jackson et al. (2007) noted the many organizational and industrial challenges in contemporary nursing workplaces that increase personal vulnerability, including heavy workloads, staff shortages, an aging workforce, bullying, and frequent organizational change and restructuring.

## Resilience

Richardson (2002) provided a review of the resilience literature and described a metatheory outlining the various conceptualizations and the evolution of research into the construct. The author draws attention to the use of different terms used across disciplines to capture resilience:

“There is a force within everyone that drives them to seek self-actualization, altruism, wisdom, and harmony with a spiritual source of strength. This force is resilience, and it has a variety of names depending upon the discipline” (2002, p. 313).

Indeed, over the years numerous conceptualizations of resilience have been proposed and studies have been conducted with vastly different groups of people (e.g., children, elderly, military, bereaved, sporting champions) and from a variety of different discipline perspectives (e.g., physics, religion, psychology, nursing). Early research in the area tended to view resilience as being determined by fixed personality traits (e.g., hardiness) that would determine how an individual responded in the face of adversity (e.g., Rutter, 1979; Garmezy et al., 1984). As research into resilience evolved, less emphasis was placed on the notion of resilience as a static phenomenon with more recognition being given to the dynamic and multifaceted processes involved in resilience (Van Vliet, 2008; Fletcher and Sarkar, 2013). Researchers have acknowledged that, rather than a single fixed personality attribute explaining resilience, there is in fact evidence for a complex array of psychological factors. For example, Fletcher and Sarkar (2012) studied Olympic athletes and found that positive personality, motivation, confidence, focus, and perceived social support all interacted to influence the stress–resilience–performance relationship.

There is considerable overlap between resilience and other psychological constructs. Coping has been regarded as a key psychological attribute or component of resilience. Gillespie et al. (2007) conducted a content synthesis of the psychology, psychiatry, and nursing literatures and concluded that coping, self-efficacy, and hope are defining attributes of resilience. Bonanno (2004), on the other hand, argued that coping is distinct from resilience and refers more generally to how people respond to or regulate stress.

Despite continued debate as to the key components of resilience, there is growing agreement regarding the importance of the environment and systemic factors in contemporary views of resilience. Lowe (2013, p. 54) theorized that resilience includes two main elements—personal factors and environmental-systems factors, and that it is the interaction between the two that fosters resilience. Similarly, when considering how to operationalize resilience, it has been argued that a suitable theoretical framework should take an interactionist approach in order “to understand and evaluate the way individuals interact with their environment” (Pangallo et al., 2015, p. 2).

While there are many definitions of psychological resilience within different contexts such as survivors of child abuse (Bonanno, 2004), resilience to loss (Mancini and Bonanno, 2009) and shame and resilience in adulthood (Van Vliet, 2008), resilience may be regarded as the product of multiple components that emphasize an individual's interaction with their environment. For instance, Windle (2011) produced a comprehensive conceptualization of resilience that includes three main components: (a) the presence of *significant* stress that carries substantial threat of a negative outcome, (b) individual and environmental resources that facilitate positive adaptation, and (c) positive adaptation or adjustment relative to developmental life stage. Consistent with this view, the unified theoretical model presented in this paper endeavors to address, in an integrated manner, a holistic perspective of relevant predictors of nursing related outcomes taking into account both individual (as identified by Rees et al., 2015) and environmental factors.

### Psychological resilience among health professionals

Rees et al. (2015) developed a model that identified the key constructs for understanding psychological resilience in the workplace for health professionals. The key constructs were self-efficacy, coping and mindfulness, with neuroticism as the mediating variable. Neuroticism, is a fundamental personality trait characterized by the tendency to experience enduring negative emotional states such as anxiety and depression (Sutcliffe and Vogus, 2003).

#### Self-efficacy

Self-efficacy is an individual's belief in his/her own ability to perform a specific task (Bandura, 1977; Garcia-Dia et al., 2013). More specifically, in the context of nursing, it is confidence in knowledge, skills, and decision-making in every day practice and the ability to deal with change and problem solving (Gillespie et al., 2007).

#### Coping

Coping is “a process of adjustment following an adverse event” (Rees et al., 2015, p. 4). Coping maybe emotion- or problem-focused. Shin et al. (2014) suggested the function of emotion-focused coping is to reduce the stressful emotions, whereas problem-focused coping aims to change the distressed person–environment relationship by aiming to solve the problem causing the distress. This process involves generating alternative solutions, and/or following an action plan. In a nursing context, problem-focused coping could include the ability to manage a constantly changing work environment (Gillespie et al., 2007) and the ability to manage unpredictable workloads.

#### Mindfulness

Mindfulness is a trait-like tendency (Rees et al., 2015) that involves focusing fully on an experience occurring in the present in an accepting or non-judgmental way (Baer et al., 2006). Some view mindfulness as a mental state and others view it as a set of skills and techniques (Brown et al., 2007). Hülsheger et al. (2013) suggest that mindfulness at work can reduce emotional exhaustion and enhance job satisfaction. Mindfulness is important if nurses are to detach themselves from highly charged emotional situations and reflect, learn and move on.

## Model development

### Literature search for key factors

The purpose of this research was to develop a theoretical model that explains the environmental factors in the workplace that promote nurses' resilience. To date, there are few models to guide research and organizational practice in this area (Sutcliffe and Vogus, 2003; Lowe, 2013; Pangallo et al., 2015), and none presents a theoretical model that unifies the individual and environmental factors that promote resilience. The development and use of a unified theoretical model would likely enable leaders of health care organizations to identify and correct processes within their workplaces that undermine resilience in their workforce. Nursing, in particular, represents more than half of all registered practitioners in the health workforce within Australia. The Australian health care sector as well as many international health care sectors, is facing a significant nursing workforce shortage into the future [Australian Institute of Health and Welfare (AIHW), 2014; Health Workforce Australia (HWA), 2014]. Building nurses' resilience to complex and stressful practice environments is necessary to cultivate neophyte nurses and retain skilled nurses in the workplace thereby ensuring safe patient care for the future.

To build new theory based on previous work by Rees et al. (2015) on the constructs of personal workforce resilience, it was necessary to gain an understanding of the different workplace factors that contributed to supporting nurses' psychological resilience to workplace stressors. To enhance understanding, a workplace resilience framework (Tables 1–**6**) was developed using the Walker and Avant (2011) literary synthesis model, which comprised several steps. First, a search and review of the relevant literature, followed by the alignment of the literature with the resilience constructs identified by Rees et al. (2015). To enable data extraction, a comprehensive review of the literature was undertaken to acquire new insights into supportive workplace environments that build resilience. Inclusion criteria were articles published in English within the last 10 years. Themes were then identified and from these themes six concepts emerged as explained later under the section on method of building the unified theoretical framework.

**Table 1:** **Support–Professional**.

**Organizational concepts: Definition**	**Environmental factors for: Support–Professional**
Professional Support is the workplace policies and structures that enable nurses to act ethically, respectfully, and benefit patient care. *(Professionalism)*	Lines of communication are explicit at both unit and organizational level. Receptive, responsive understanding, supportive leadership at unit, and organizational level. Timely access to senior clinical support/line manager for guidance on ethical dilemmas. Respectful and receptive working relationships with colleagues that encourages questioning and innovation.

**Figure 1:**
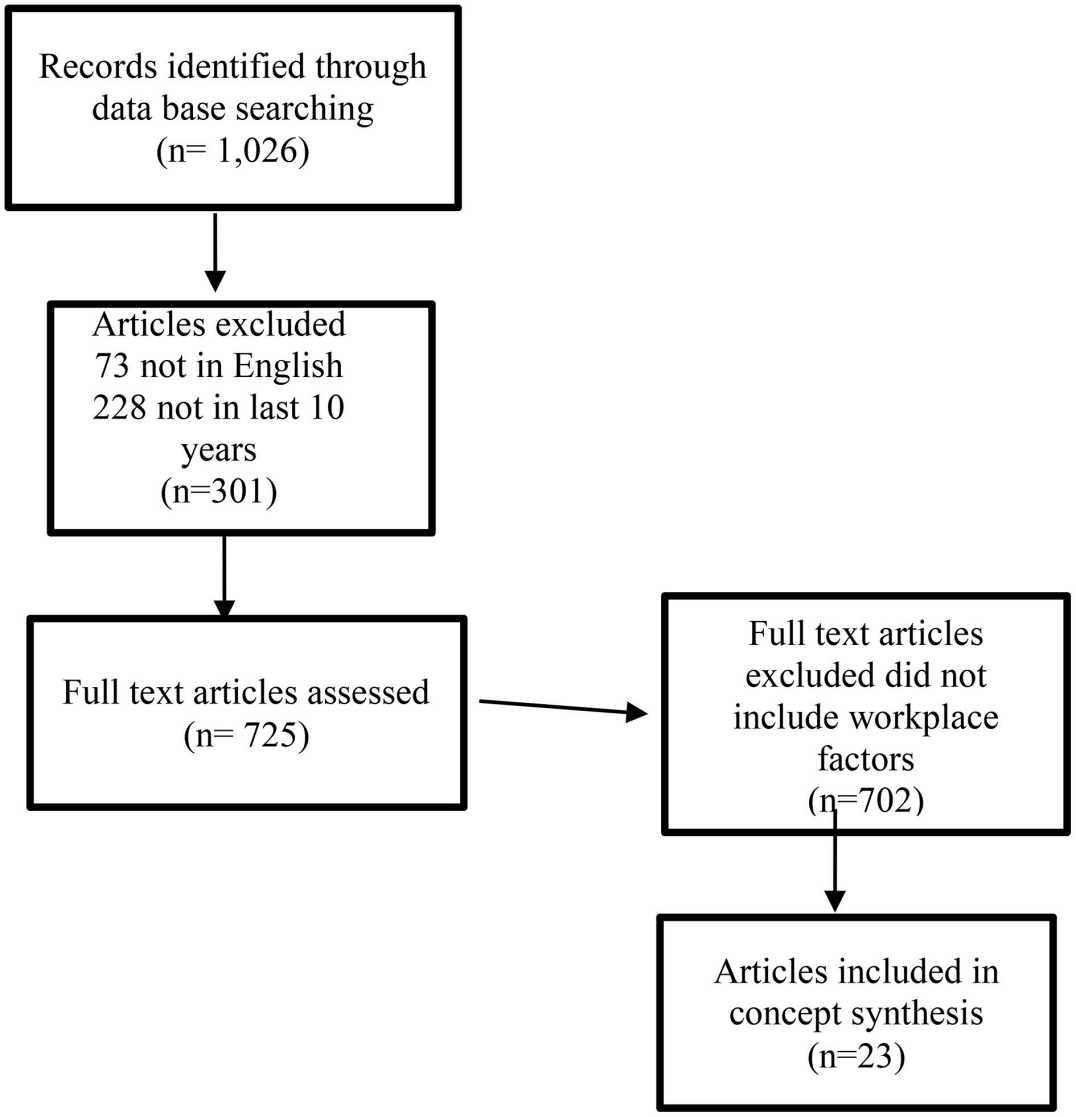
**Search results for workplace resilience and nurses (Prisma, 2009; http://www.prisma-statement.org/)**.

A search of peer reviewed literature using PubMed and CINAHL was undertaken. For the PubMed search, the search terms Nurs^*^[tw] OR jsubsetn[text] and Resilien^*^[tw] were used yielding 864 citations with 809 of these written in English; 621 citations were published in the last 10 years. For the CINAHL search, the search terms used were Nurs^*^ [Ab] and Resilien^*^ [TI] yielding 162 citations, 144 written in English; 104 were written in the last 10 years. All titles and abstracts from January 2004-March 2014 were checked manually for relevance. In PubMed, 124 sources were identified as relevant. Within these, 22 sources identified environmental factors within practice settings relevant to individual resilience. CINAHL yielded one additional relevant source totalling 23 fit for purpose sources (see Figure 1: Search results).

The literature identified a considerable number of environmental workplace factors that were considered important in enabling a supportive work environment for nurses. These workplace factors were relational, mentoring, clinical supervision, education and training, staffing levels, personal safety, and self-care (Hodges et al., 2008; Mealer et al., 2012a; Rickard et al., 2012; McCann et al., 2013; Wallbank, 2013).

Relational factors were one of the leading themes from the literature that enabled resilient work environments. Relational factors in the work environment included a range of collegial interactions such as: fostering collaborative inter-professional relationships within the team environment, transparent, open lines of communication throughout the work environment, accessible senior staff, feeling valued by the organization and opportunities to contribute to decision making (Veninga, 2007; Warelow and Edward, 2007; Lowe, 2013; McCann et al., 2013; Huddleston, 2014; Nowrouzi et al., 2015). Informal mentoring through access to positive role models was also noted to inspire and build staff confidence, particularly for new staff (Warelow and Edward, 2007; Mealer et al., 2012b; Garcia-Dia et al., 2013).

Access to formal mentoring and clinical supervision were workplace strategies frequently mentioned in the literature as initiatives that not only supported staff but also improved patient outcomes (Hodges et al., 2008; Mealer et al., 2012a; Rickard et al., 2012; McCann et al., 2013; Wallbank, 2013).

Training and education was regularly identified as a workplace factor that supported staff to improve their self-efficacy (American Association of Critical Care Nurses (AACN), 2005; Veninga, 2007; Warelow and Edward, 2007; Hodges et al., 2008; Mealer et al., 2012a; Rickard et al., 2012; McAllister, 2013). Nurses felt better prepared and more clinically confident to undertake the complex care of patients assigned to them.

In addition to education support, appropriate rostering, adequate staffing as well as clarification of role expectations were factors in the literature that influenced nurses' ability to cope with the workload. Simple strategies included making sure staff took their tea breaks and had the time allocated for them to attend training programs (Rickard et al., 2012; Huddleston, 2014).

Two workplace factors that emerged more recently in the literature relate to nurses' feelings of personal and psychological safety and promotion of self-care (Grafton et al., 2010; Rickard et al., 2012; Garcia-Dia et al., 2013; Lowe, 2013; McCann et al., 2013). A positive response to personal and psychological safety of nurses was linked to workplace cultures where managers acted through policy development and implementation of strategies to prevent workplace violence, and help staff cope with critical incidents. This could include strategies such as mindfulness training (Shapiro et al., 2005) and the provision of access to a quiet room after a traumatic incident on the ward.

The concept of self-care was raised in the literature. Nurses were encouraged to maintain their work-life balance, to have supportive social networks, actively manage the number of shifts or hours worked and proactively access counseling through employer assistance programs (McAllister and McKinnon, 2009; Grafton et al., 2010; Hayes et al., 2012; Rickard et al., 2012; Garcia-Dia et al., 2013; Lowe, 2013; McCann et al., 2013).

From the literature review, the most frequently identified workplace environmental factors were identified, extracted and clustered in alignment with key constructs for psychological resilience—self efficacy, coping, and mindfulness. Workplace environmental factors of similar or broader nature were further reduced into higher-order factors within each key construct for psychological resilience. Throughout this process, the research team consistently reviewed the logic applied to the literature synthesis via a decision-making map developed by the lead researchers. They tracked the steps of identifying and clustering the workplace environmental factors under the key constructs including feedback and verification from the broader research team. Once the workplace factors under each key resilience concept were reduced as much as possible and themed, six major organizational concepts emerged that related to a positive workplace environment and formed the foundation of the theory. Three of the organizational concepts related to nursing staff support within the workplace environment and three of the organizational concepts related to nursing staff development within the workplace environment. The environmental factors listed next to each organizational concept suggest key organizational policies, procedures and systems that, if implemented, would have a direct relationship to psychological resilience of nursing staff.

The draft workplace resilience framework was then subject to a rigorous review process. Six very experienced senior nurses, nursing academics and other resilience researchers were identified by the research team and invited to provide a review. The framework was forwarded to the six reviewers separately for individual consideration of the organizational concepts and environmental factors for feedback to the lead researcher. Comments were collated and a refined framework was presented for further discussion and analysis at a face- to-face meeting of the reviewers. This discussion confirmed the extent to which the identified environmental factors would adequately sample the defined organizational concepts, thus supporting face and content validity of the framework.

## Health service workplace environmental resilience model

Two overarching organizational concepts emerged—*Support* and *Development*. For the purpose of this theory, ***Support*** is defined as those workplace interventions that nurture and enable nurses to withstand workplace pressures while ***Development*** is defined as those workplace interventions that empower nurses to enhance their professional, practice and personal potential. Further, within each organizational concept there are three domains—Personal, Practice, and Professional. The ***Personal*** domain encompasses nurses' individual well-being. The ***Practice*** domain comprises the discipline-specific skills, capabilities, and competencies of a profession. The ***Professional*** domain is about an ideal of service which includes life-long learning and adherence to ethical behavior.

It follows then that the organizational concept of *Support* and the three domains, Professional Support (Table 1), Practice Support (Table 2), and Personal Support (Table 3) would nurture and enable nurses to withstand workplace pressures and thus contribute to their psychological resilience in the workplace (self-efficacy, coping, mindfulness).

**Table 2:** **Support–Practice**.

**Organizational concepts: Definition**	**Environmental factors for: Support–Practice**
Practice Support is workplace processes that enable nurses to deliver competent, patient-centered nursing care. *(Competence)*	Role expectations explicit at both unit and organizational level but not restrictive. Patient allocation matched to the individual skills and experience. Nurse-patient ratio and systems for staff allocation consider experience and complexity of care. Timely access to experienced clinical support for guidance on practice dilemmas. Easily accessible contemporary and sensible clinical policies and procedures. Essential resources including equipment that is available and working correctly. Organization supports respectful inter-professional collaboration that facilitates safe patient care.

**Table 3:** **Support–Personal**.

**Organizational concepts: Definition**	**Environmental factors for: Support–Personal**
Personal Support is the health and safety workplace practices that enable nurses to feel connected, safe and keep well. *(Well-being)*	Unit and organizational culture that role models kindness and positive staff behaviors. Regular staff meetings that address sources of stress and seek collaborative solutions. OHS policies that maximize physical and psychological well-being including workplace violence control. Meal breaks planned and monitored to ensure they can be taken. Time out opportunities available after challenging situations to practice mindfulness strategies. Roster system that facilitate rest and engagement with family, friends and community. Access to early assistance for anxiety states. Access to Employment Assistance Programs. Access to annual/long service/ personal leave encouraged when time-out for self- care required. Physical spaces provided conducive to mindfulness breathing exercises and short meditations.

Likewise, the organizational concept of *Development* and the three domains, Professional Development (Table 4), Practice Development (Table 5), and Personal Development (Table 6) would empower nurses to enhance their professional, practice and personal potential and thus contribute to their psychological resilience in the workplace (self-efficacy, coping, mindfulness).

**Table 4:** **Development–Professional**.

**Organizational concepts: Definition**	**Environmental factors for: Development–professional**
Professional Development is the workplace policies and structures that provide opportunities for nurses to engage in reflection, career development, and lifelong learning. *(Professionalism)*	Mentoring programs available that promotes bigger picture thinking and career development planning. Performance development review processes that promote staged knowledge and skill development. Opportunities that encourage reflection on practice, feelings, and beliefs and the consequences of these for individuals/groups. Access to study leave.

**Table 5:** **Development–Practice**.

**Organizational concepts: Definition**	**Environmental factors for: Development–practice**
Practice Development is the workplace processes that provide opportunities to enhance clinical nursing practice. *(Competence)*	Continual practice development opportunities around clinical knowledge, skills, and problem-solving. Clinical supervision systems that build competence and confidence. Opportunities to debrief and learn from mistakes using an educative rather than a blaming approach.

**Table 6:** **Development–personal**.

**Organizational concepts: Definition**	**Environmental factors for: Development–personal**
Personal Development is the workplace practices that provide opportunities for nurses to develop skills that build resilience. *(Well-being)*	Learning opportunities in adaptive coping. Education and training on mindfulness and meditation skills. Learning opportunities around anxiety recognition and management.

## Discussion and limitations

Building and strengthening workplace resilience is important in stressful work environments. A number of adverse events or antecedents may carry a significant threat to nursing staff resilience, such as workplace violence, unpredictable workloads, compassion fatigue, lack of resources, workplace bullying, and lack of capacity to influence good patient outcomes (Jackson et al., 2007; Melnyk et al., 2013; Nowrouzi et al., 2015). While these events may be one-off, traumatic occasions that test the nursing staff's ability to bounce back, more commonly it is a continually negative workplace environment and culture that may culminate in staff burnout or compassion fatigue. If nurses cannot remain sufficiently resilient over time, then this ongoing vicarious trauma may lead to depression, poor physical health outcomes, higher staff turnover as well as poor patient care (Choi et al., 2011; Rickard et al., 2012). As identified by Pangallo et al. (2015, p. 1) it is important to make a distinction between chronic and acute stressors because resilience is likely to co-vary with the type and duration of a given stressor. Nurses are likely to be most exposed to chronic systemic stressors where the practice environment may not be conducive to providing a supportive workplace over a long period of time. Therefore, key workplace interventions that support nurses' resilience are important for ongoing staff retention and quality patient care (Hayes et al., 2012).

The Health Service Workplace Environmental Resilience Model (HSWERM) presents a unified framework for Support and Development across Personal, Practice and Professional domains, yielding six organizational concepts. Each of these concepts has a relationship with a range of key workplace attributes identified from the literary synthesis as influencing nurses' psychological resilience. Managers, through their leadership role, can influence organizations through interventions aligned with these key concepts. A strong relationship exists between the work environment systems/processes, the ability of the manager/senior staff to influence systems/process and staff psychological adaptation (Cho et al., 2006; McAllister and McKinnon, 2009; Choi et al., 2011; McCann et al., 2013; Roche et al., 2014). Research undertaken by Edmondson (1999, p. 377) also identified that effective team leader coaching and context support, such as adequate resources and information, appear to contribute to an environment in which team members can develop self-efficacy and trust in the team, building team psychological safety.

**Figure 2:**
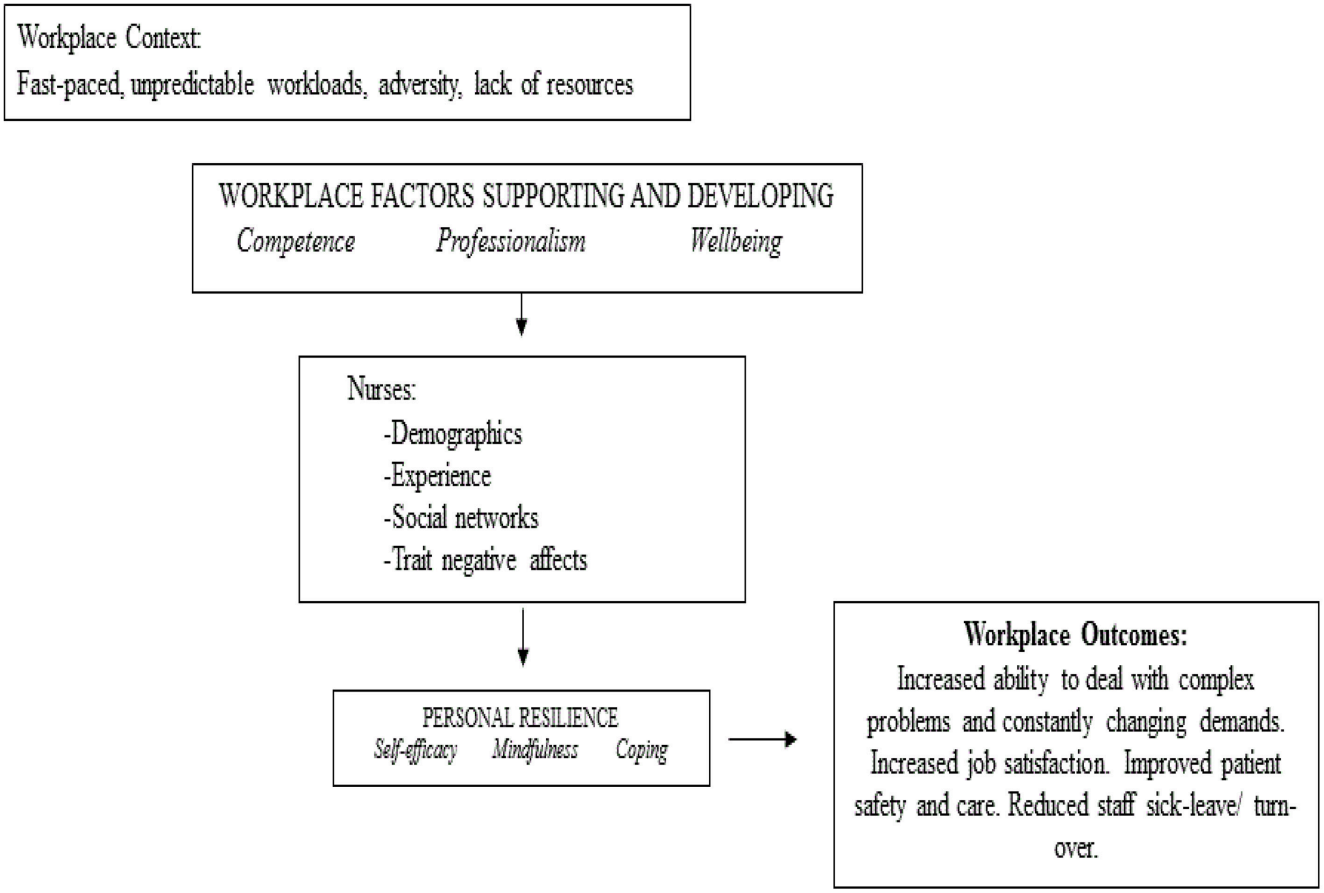
**Health service workplace environmental resilience model**. The HSWERM incorporates strategies for support and development across professional, practice, and personal domain within the workplace context, linking to the nurse, and then impacting on personal resilience and workplace outcomes.

If nursing leaders are able to implemented interventions that influence the identified key factors in the workplace environment that enable nurses to have the ability to recover, re-bound, bounce-back, adjust or even thrive following adverse events, then they will strongly influence the culture and retention of nurses into the future. As a unified theoretical model of the framework for workplace environmental resilience, the HSWERM clearly identifies that an individual's resilience can be strengthened or diminished by a range of environmental factors specific to the workplace (See Figure 2).

This approach is supported by Windle's (2011) conceptualization of resilience that emphasizes both the resources within individuals and their environment. These components are reflected in our unified theoretical framework. The next step is to undertake further research to validate, refine and test the HSWERM framework in the nursing context.

The HSWERM offers a balanced approach wherein individuals have a responsibility to maintain and build personal resilience and senior staff members in organizations have a responsibility to support personal resilience by providing a safe and supportive practice environment. Existing research shows that nurses, as well as other health professionals, are typically encouraged to engage in personal strategies to build resilience with limited attention to the role of environmental factors in the workplace (Lowe, 2013; Breen et al., 2014). While some workplaces provide workshops to encourage and assist nurses to build their resilience, these may be of limited value if the workplace environmental factors that diminish resilience are allowed to flourish. A focus on building nursing workforce resilience that blends strategies to help nurses build their own resilience (such as mindfulness workshops) whilst implementing change in nursing workplace cultures that are known to diminish resilience (such as addressing bullying) could be a powerful combination. Without a focus on workplace factors that promote resilience, individuals maybe unfairly blamed for their vulnerability to workplace stress and burnout.

The development of this theory is influenced by previous conceptualizations as well as current understandings of the workplace contexts, which are continually evolving. However, the authors have extensive experience in contemporary nursing practice. The challenge is to operationalize the model so that the factors can be examined in future research and critically reviewed as enablers of psychological resilience that lead to positive outcomes for nurses and patients.

## Conclusion

This analysis is based on a literary synthesis of nursing literature building on a previous workplace resilience model to explain theoretically the environmental factors that affect nurses' resilience in the workplace. Six organizational concepts that address nursing staff support and nursing staff development within personal, practice and professional domains were identified in the model. Healthcare leaders can consider these concepts when seeking to enhance the psychological resilience of nursing staff. These concepts underpin interventions which include mentoring, supervision, education, staffing levels, patient safety, and self-care may sound surprisingly simple/ordinary but their enactment takes strong leadership and commitment. Environmental factors that promote psychological resilience have major potential to increase staff retention and quality of patient care.

## Author contributions

LC, MS had substantial contributions to the literature review, conception, design, analysis, and development of the theory and model and the article; LC, MS, DH, CSR, LB, AW contributed to the interpretation and development of data for the design of the theory, model, and article. LC, MS, DH, CSR, LB, AW, CR, WC, RW, KC all contributed significantly to the revising of the article contributing their important intellectual content to the final version. All authors approved the final version to be published. Final approval of the version to be published.

### Conflict of interest statement

The authors declare that the research was conducted in the absence of any commercial or financial relationships that could be construed as a potential conflict of interest.
